# Supplementary material for: Characterization of a Common S Haplotype BnS-6 in the Self-Incompatibility of Brassica napus
Source: Plants (Basel). 2021 Oct 15;10(10):2186. doi: 10.3390/plants10102186 (PMC8537745; doi:10.3390/plants10102186)
Supplement: Supplementary file 1 [file plants-10-02186-s001.zip › Supportment Figure.pdf]

```
BnSCR1300   ...........GTTGGATTGAAGAT...GGCCTAAGTGTTTTTTAA........ACCAGA    38
BnSCR6      ....TTTTAGTGTTGGATTGGAGAT...AGCCTAAGTGTATTTCAA........AGGAGA    45
BnSCR7      TTTGTTATTATGTAAGGTTACCAATCAAGGTCTAAGTGTATTTTGAGTGATGACACGAGA    60
Consensus              gt  g tt    at    g ctaagtgt ttt  a       a   aga

BnSCR1300   AGAGCTCCGCAGGAAAAGAAGAATTGGATGAGGAAGTTACACACACGAATAAGGTGTGAC    98
BnSCR6      AGAGCTCCGCAGGAAAAGAAGAATAGGATGAGGAAGGTACACACACGAATAAGGTGTGAC   105
BnSCR7      GGAGCTATGCAGAAAAAGAAGAACAGGGTGAGGAA.....ACACACGAATAAGGTGTGTC   115
Consensus    gagct  gcag aaaagaagaa  gg tgaggaa     acacacgaataaggtgtg c

BnSCR1300   CCGAATTGTTTACGTGTAAAATAGGCAATTAAGTGCAAGATGTGAAGGAAAAAAAATATA   158
BnSCR6      CCGAATTGTTTACGTGTAAAATAGGCAATTATGTGCTAGATGCGAAGAAAAA......CA   159
BnSCR7      ....AATGTTTACGTGTGAAATAGGCAATTAAGTGCAAGATCTGTGAAGAAAAAAAATATA  171
Consensus       a tgtttacgtgt aaataggcaatta gtgc agat  g     aaa       a

BnSCR1300   TATATATAGTAATACAAGCAATAACATTCTATAAAAAAGCGAAAATCTTATATACTCATA   218
BnSCR6      TATATATAGTAATACAAACAATAACATTCTATAAAAAAGCGAAAATCTTATATACTCATA   219
BnSCR7      TATATATC.TAGAGTAAACAATAACATTCTACAGAAA.GCGACGATCTTATATATTCAGA   229
Consensus   tatatat  ta    aa caataacattcta a aaa gcga  atcttatata tca a

BnSCR1300   AGTCATGAGATATGCTACTTCTATATATACATTTTTAACAAATATACACTACTTATGTTT   278
BnSCR6      AGTCATGAGATATGCTACTTCTATATATACATTTTTAACAAATATACACTACTTGTGTTT   279
BnSCR7      AGTCATGAGATATGCTACTTCTATATTTTTTTTTTTAACAAAGATACACTACTTGTGTTT   289
Consensus   agtcatgagatatgctacttctatat t   tttttaacaaa atacactactt tgttt

BnSCR1300   CATATTTTTGATTTTGACATATGTTCAAGGTAAACTATATCAATAACTTTCCCCCTTTTA   338
BnSCR6      CATATTTTTGATTTTGACATATGTTCAAGGTAAACTATATCAATAACTTTCCCCCTTTTA   339
BnSCR7      CATATTTTTGACTTTGACATCTGTTCAAGGTAAACTATATCAATAACTTTTCCCCTCTTA   349
Consensus   catattttga tttgacat tgttcaaggtaaactatatcaataacttt cccct tta

BnSCR1300   TGGACGCTTTAGGATTTTTCTTACCTAATTGCAATTCATAATTTTTTGTTAATTTAAAGC   398
BnSCR6      TGGACGATTTAGGATTTTTCTTACCTAATTGCAATTCATA.TTTTTTGTTAATTTAAAGC   398
BnSCR7      TTGACGATTTAGGATTTTTCTTACCCAATTGCAATTCATAATTTTT..TTTATTTAAAGC   407
Consensus   t gacg tttaggatttttcttacc aattgcaattcata ttttt  tt atttaaagc

BnSCR1300   ACTAGATGTGGGAGCTTGGAAATGCCCTGAAGGCATCGTCTATCCGAGTCCTATCTCAGG   458
BnSCR6      ACTAGATGTGGGAGCTTGGAAATGCCCTGAAGGCATCGCCTATCCGAGTCCTATCTCAGG   458
BnSCR7      ACTAGATGTGGGAGCTAGGAACTGCCCTGAAGGCATCGCCAAATCGAATGATGTCATAGG   467
Consensus   actagatgtgggagct ggaa tgccctgaaggcatcg c a  cga t  t tc  agg

BnSCR1300   AAGGTGCATTAATTCCAGGAGCACAGAGTGTAAAAAACACTATGAAGTTGAGGGACAGAA   518
BnSCR6      AAGATGCTTTAATTCCAGGAGCACAGAGTGTAAAAAACACTATGAAGTTGAGGGACATAA   518
BnSCR7      AACATGCTTAAATACCAAGAGCAGAGACTGTCAAAAACACTTCG.........GACCGAA   518
Consensus   aa  tgc t aat cca gagca aga tgt aaaaacact  g         gac  aa

BnSCR1300   TGTTACTAATTGCCGTTGTGATACTTATAGCATGCAAAATCCTGCGAGGATTACTTGCTA   578
BnSCR6      TGTTACTAATTGCCGTTGTGATACTTATAGCATGCAAAATCCTGCGAGGATTACTTGCTA   578
BnSCR7      TGTTACTAATTGCCTTTGTTATCCTTTTAGCACGCATAATCGTGTGAGGATTACTTGCTA   578
Consensus   tgttactaattgcc ttgt at ctt tagca gca aatc tg gaggattacttgcta

BnSCR1300   CTGTTGCAAAGTTAAATCATAATTGATCAACGAAACATCCAGAGACGGTTAC           630
BnSCR6      CTGTTGCAAAGTTAAATCATAATTGATCAACGAAACATCCAGAGACGGTCAC           630
BnSCR7      CTGTTGCAAAGTTAAATCATAATTGATCAACGAAACATCCAGATACGATTAC           630
Consensus   ctgttgcaaagttaaatcataattgatcaacgaaacatccaga acg t ac
```

Figure S1. Collinear comparison of *SCR* genomic sequences of *BnS-1300*, *BnS-6* and *BnS-7*. The SCAR marker primers were underlined with black. The qPCR primers were highlighted in green, and the blue box indicated the mutant position. The red box represented the initiation codon and termination codon, and the gray box represented the intron.

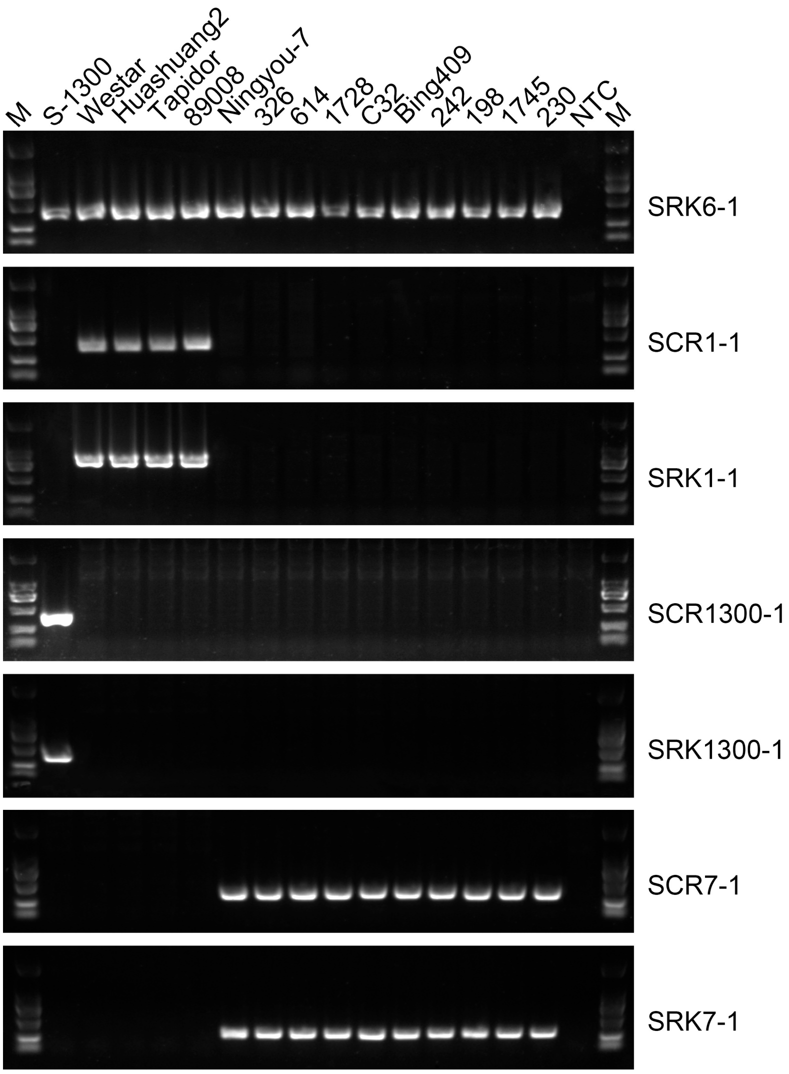

Figure S2. PCR fragments amplified from 'S-1300' and 14 *B. napus* SC lines using SCAR markers. NTC: no template control. M: DNA marker, from top to bottom, the size of band was 2,000, 1,000, 750, 500, 300, and 200 bp, respectively.

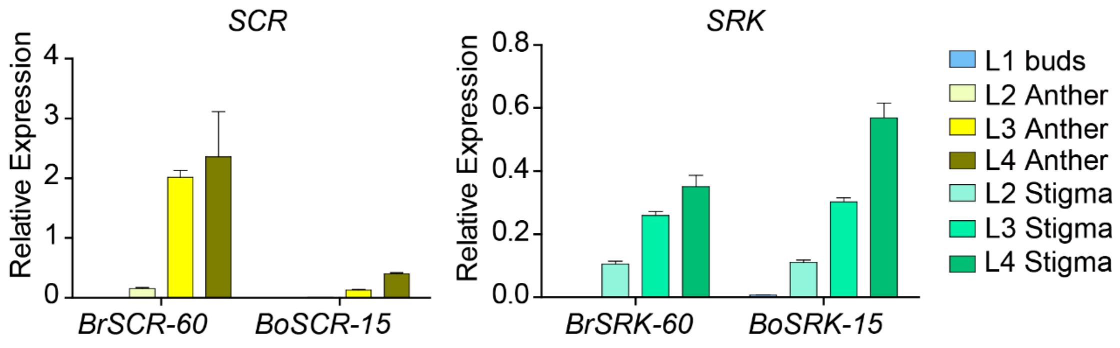

Figure S3. Expression analysis of *SCR* and *SRK* in the flower tissues of *B. rapa* and *B. oleracea*. The picture on the left shows the expression of *SCR* in the flowers of *B. rapa* and *B. oleracea* respectively. *BrSCR-60* is expressing in the anthers of different developmental stages of *B. rapa* 'BrHB'. The right picture shows the expression of *SRK* in the stigma of *B. rapa* and *B. oleracea* respectively. *BrSRK-60* is expressing in the stigma of different development stages of *B. rapa* 'BrHB'. *BoSRK-15* is expressing in the stigma of different developmental stages of *B. oleracea* 'BoRBR'.

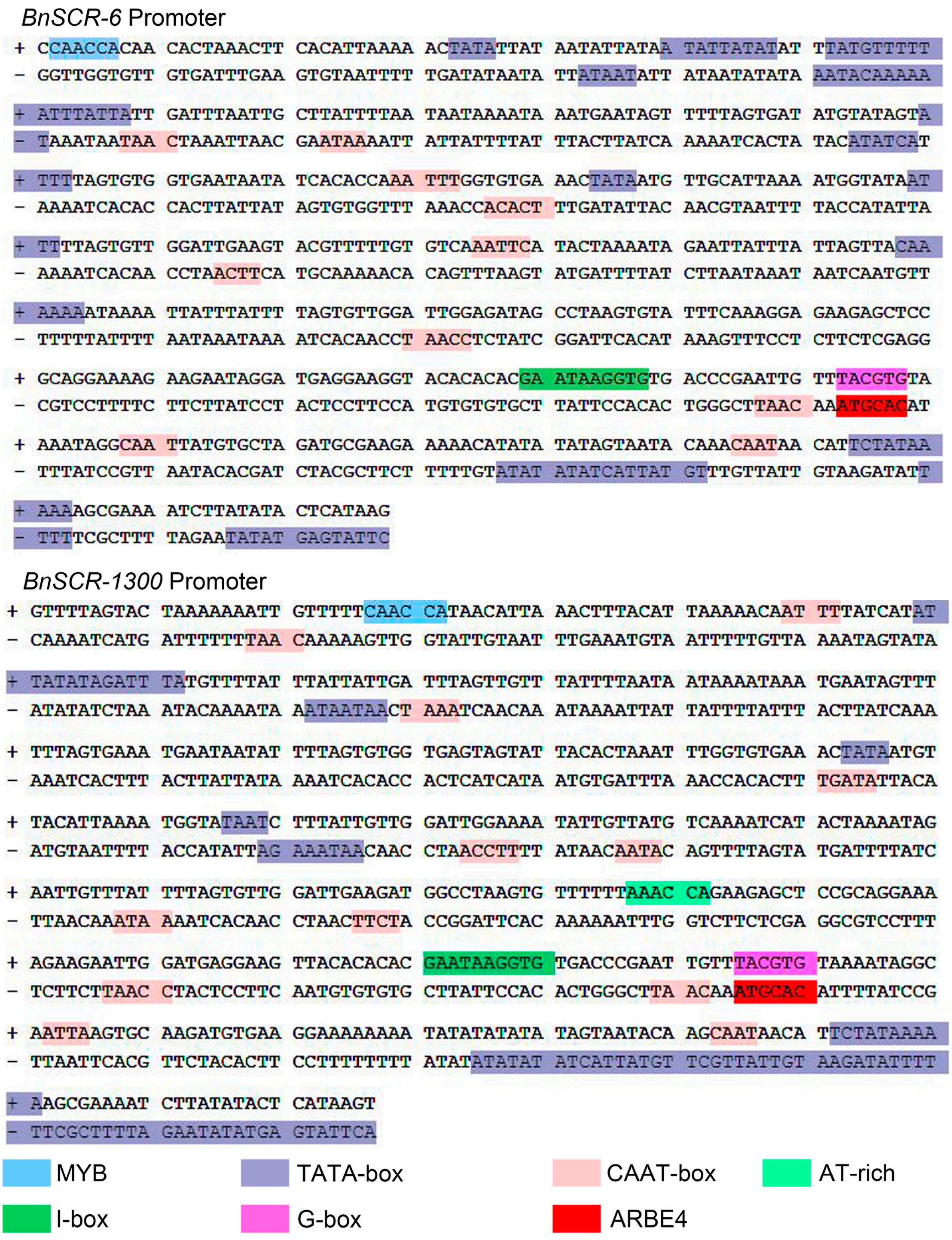

Figure S4. *cis*-elements prediction in the promoters of *BnSCR-6* and *BnSCR-1300*
