# Supplementary material for: Characterization of a Common S Haplotype BnS-6 in the Self-Incompatibility of Brassica napus
Source: Plants (Basel). 2021 Oct 15;10(10):2186. doi: 10.3390/plants10102186 (PMC8537745; doi:10.3390/plants10102186)
Supplement: Supplementary file 1 [file plants-10-02186-s001.zip › Supportment Table1.pdf]

Table S1. Primers used in this study

Primer name	Sequence (5'-3')	Use
SRK6-1-F	ATTCGATTGTGTTTCAGGCTC	
SRK6-1-R	TCGACATGGTGATTTGGTTC	
SCR1-1-F	TCTGAATCATGAAATCTGCTGT	
SCR1-1-R	TTAAAAGGATTCTGCAAAGTTCT	
SRK1-1-F	CTTCTGATCATGTTTTGCCTCTG	
SRK1-1-R	CGAGATCTTTGGGACCATATTTC	
SCR1300-1-F	TGAAGATGGCCTAAGTGTTTTTAAACC	SCAR marker
SCR1300-1-R	GCTCCTGGAATTAATGCAC	
SRK1300-1-F	GTTCGGTTCACTACGTAAAAAC	
SRK1300-1-R	CTGAGGAATAATAGGAGATACG	
SCR7-1-F	GTAAACAATAACATTCTACAGAAAGCGACG	
SCR7-1-R	CAAAGGCAATTAGTAACATTCGGTCCGA	
SRK7-1-F	AGTGGGATCTGCAAACAGTTG	
SRK7-1-R	GGGCTAATGTATCATATATATGACTTGACC	
SRK7-F	CGGTTTAGAATCATCCATAG	
SRK7-R	CATCGTCACGCCTAGAAT	
SRK6-F	GAATGGCAAGGATCTTTGGACGG	
SRK6-R	TAGACCTTGACCTTCTTTCCAATTC	
SRK1300-F	GAATGGCAAGGATCTTTGGACAA	
SRK1300-R	TAGACCTTGACCTTCTTTCCAATTT	
SRK1-F	GAAGACAATCTTCTAAGCTGTGC	
SRK1-R	CACACAAGAGACCAATTTGTATGC	
SCR7-F	CTGAAGGCATCGCCAAATCG	qRT-PCR for *SCR* and *SRK* in *B. napus*
SCR7-R	ATCGTATCTGGATGTTTCGTTG	
SCR6-F	ATATACATTTTTAACAAATATACACTACTTG	
SCR6-R	TGTGCTCCTGGAATTAAAGCGT	
SCR1300-F	CTTCTATATATACATTTTTAACAAATATACACTACTTA	
SCR1300-R	ACACTCTGTGCTCCTGGAATTAATGCTC	
SCR1-F	CATCGTTTCAGGTCATATTCAAGAAG	
SCR1-R	AGAATTTGTGCATTCGCAACG	
Actin7-F	CTATCCTCCGTCTCGATCTCGC	
Actin7-R	CTTAGCCGTCTCCAGCTCTTGC	
SCR-6-R	TACTGGGTCTTATTAATTAGTCTTTGAGT	Amplification of *BnSCR-6* genomic sequence and 5'-
SCR-6P1-F	CTAACTATTTCCTTTTTAAGCACG	
SCR-6P2-F	TTCCAACCACAACACTAAACTTCAC	

SCR-6P3-F	GTGATATGTATAGTATTTTAGTGTG	deletion promoter constructs of *BnSCR-6*
SCR-6P4-F	TGTTGGATTGAAGTACGTTTTTG	
SCR-6ProR	GACTTATGAGTATATAAGATTTTCGC	
SCR-1300P1-F	ACGAATAAGGTTTATCCGAATGTTT	5'-deletion promoter constructs of *BnSCR-1300*
SCR-1300P2-F	CAAAAACATCTCCAATACATAACAG	
SCR-1300P3-F	TAATAAAATAAATGAATAGTTTTTTAGT	
SCR-1300P4-F	TTATTGTTGGATTGGAAAATATTGTTA	
SCR1300-ProR	GACTTATGAGTATATAAGATTTTCGC	
47-684proF	CGAGCTCGACACACCATCACCACTTCTTT	*BnSCR-6* overexpression
47-1860proF	CGAGCTCAGCTTCACTCTTGGACTGTC	
47-proR	ACGCGTCGACGATTCAGAAAAGTGATAAAAGATTC	
SCR6CDS-F	ACGCGTCGACATGAGATATGCTACTTCTATATATAC	
SCR6CDS-R	GGGTAACCTTATGATTTAACTTTGCAACA	

F, forward primer; R, reverse primer.
